# Betaine in Inflammation: Mechanistic Aspects and Applications

**DOI:** 10.3389/fimmu.2018.01070

**Published:** 2018-05-24

**Authors:** Guangfu Zhao, Fang He, Chenlu Wu, Pan Li, Nengzhang Li, Jinping Deng, Guoqiang Zhu, Wenkai Ren, Yuanyi Peng

**Affiliations:** ^1^College of Animal Science and Technology, Southwest University, Chongqing, China; ^2^Guangdong Provincial Key Laboratory of Animal Nutrition Control, College of Animal Science, Subtropical Institute of Animal Nutrition and Feed, South China Agricultural University, Guangzhou, Guangdong, China; ^3^Jiangsu Co-Innovation Center for Important Animal Infectious Diseases and Zoonoses, Joint International Research Laboratory of Agriculture and Agri-Product Safety of Ministry of Education of China, College of Veterinary Medicine, Yangzhou University, Yangzhou, China

**Keywords:** betaine, oxidative stress, endoplasmic reticulum, inflammation, obesity

## Abstract

Betaine is known as trimethylglycine and is widely distributed in animals, plants, and microorganisms. Betaine is known to function physiologically as an important osmoprotectant and methyl group donor. Accumulating evidence has shown that betaine has anti-inflammatory functions in numerous diseases. Mechanistically, betaine ameliorates sulfur amino acid metabolism against oxidative stress, inhibits nuclear factor-κB activity and NLRP3 inflammasome activation, regulates energy metabolism, and mitigates endoplasmic reticulum stress and apoptosis. Consequently, betaine has beneficial actions in several human diseases, such as obesity, diabetes, cancer, and Alzheimer’s disease.

## Introduction

**Figure 1:**
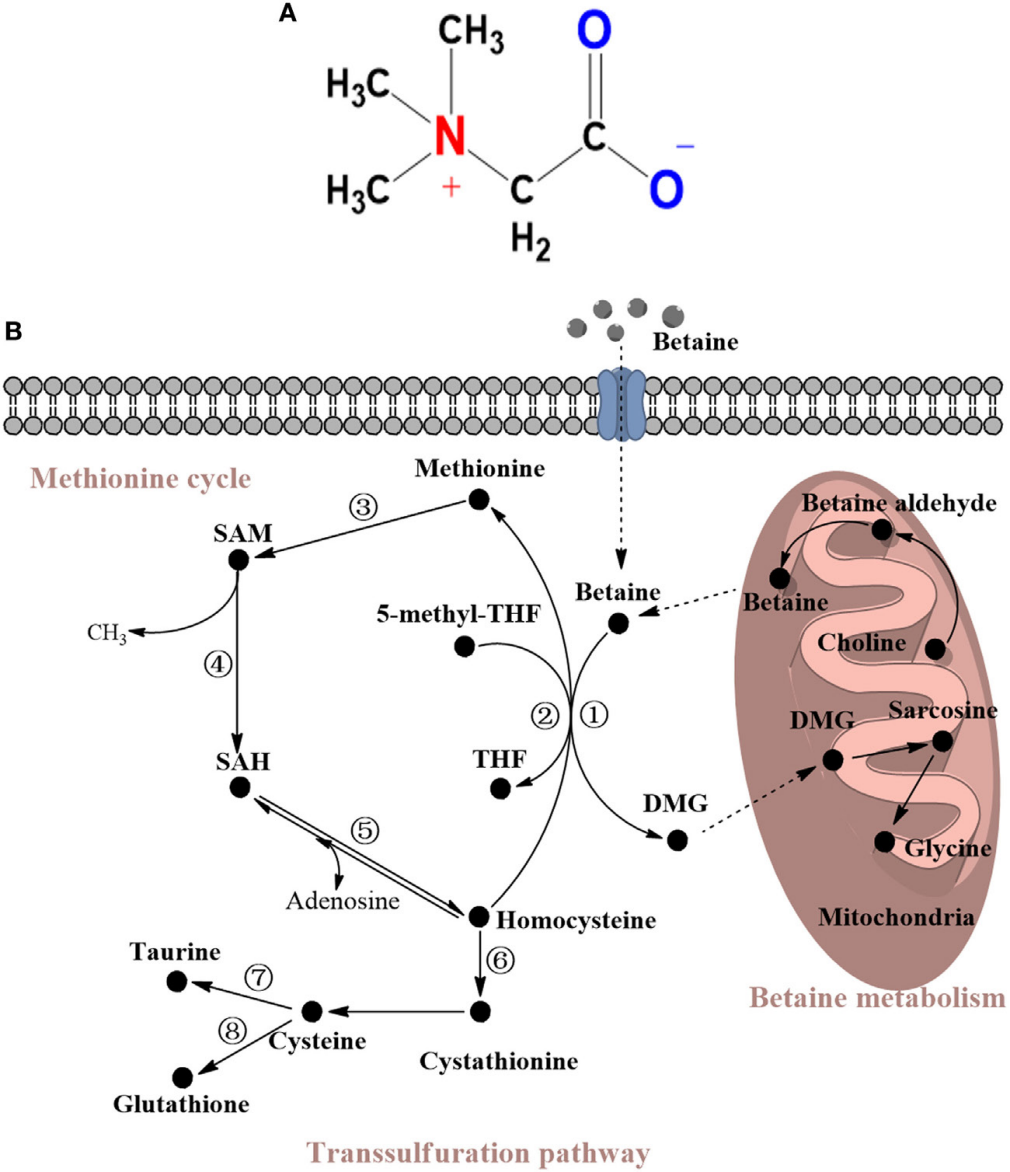
(**A**) Molecular structure of betaine. (**B**) Metabolism of betaine and related sulfur amino acids (SAAs). Betaine is a substrate of choline and can be converted to DMG *via* demethylation to ultimately become glycine. Most of these reactions occur in the mitochondria. The demethylation reaction converts homocysteine to methionine and can be replaced by 5-methyl-THF, which can catalyze methylation to form THF. Then, methionine is successively converted to SAM and finally to homocysteine to form the methionine cycle. Homocysteine can also go through the transsulfuration pathway to form cystathionine, cysteine, taurine, or glutathione. The enzymes mentioned in this review are shown and marked in the cycle with individual numbers. 1. Betaine-homocysteine methyltransferase (BHMT); 2. Methionine synthase (MS); 3. Methionine adenosyltransferase (MAT); 4. SAM-dependent methyltransferases; 5. S-adenosylhomocysteine hydrolase; 6. Cystathionine β-synthase (CBS); 7. Cysteine dioxygenase (CDO); 8. γ-glutamylcysteine synthetase (GCS). THF, tetrahydrofolate; SAM, S-adenosyl-L-methionine; SAH S-adenosyl-L-homocysteine; DMG, N,N-dimethylglycine.

Betaine is a stable and nontoxic natural substance. Because it looks like a glycine with three extra methyl groups, betaine is also called trimethylglycine (1). In addition, betaine has a zwitterionic quaternary ammonium form [(CH3)3N+ CH2COO−] (Figure 1). In the nineteenth century, betaine was first identified in the plant *Beta vulgaris*. It was then found at high concentrations in several other organisms, including wheat bran, wheat germ, spinach, beets, microorganisms, and aquatic invertebrates (2). Dietary betaine intake plays a decisive role in the betaine content of the body. Betaine is safe at a daily intake of 9–15 g for human and distributes primarily to the kidneys, liver, and brain (2). The accurate amount of betaine intake generally relies on its various sources and cooking methods (3). Besides dietary intake, betaine can be synthesized from choline in the body. Studies report that high concentrations of betaine in human and animal neonates indicate the effectiveness of this synthetic mechanism (4, 5).

Regarding its biological significance, on the one hand, betaine is a vital methyl group donor in transmethylation, a process catalyzed by betaine-homocysteine methyltransferase (BHMT). This reaction catalyzes homocysteine to form methionine and occurs primarily in the liver and kidneys (6). On the other hand, betaine is an essential osmoprotectant, primarily in the kidneys, liver, and brain, and large amounts of betaine can accumulate in cells without disrupting cell function; importantly, this role of betaine protects cells, proteins, and enzymes under osmotic stress (7).

Recently, several studies have focused on various natural compounds proven to be effective against many diseases. For example, Geng and colleagues found that mulberrofuran G has anti-hepatitis B virus activity (8). Interestingly, in Southeast Asia, water extracts of *Lycium chinensis*, which contains a high concentration of betaine, were used as a traditional oriental medicine to treat liver disorders (9). These findings indicate that the function of betaine, a natural compound, has become a hot topic because of its anti-inflammatory effects on diseases, such as nonalcoholic and alcoholic fatty liver disease (NAFLD and AFLD) and diabetes (10–12). This paper summarizes the role of betaine in physiological functions, anti-inflammatory mechanisms, and human diseases.

## Physiological Functions of Betaine

As many studies show, cells from bacteria to vertebrates absorb betaine as an osmoprotectant; animals can rapidly absorb betaine through the duodenum of the small intestine (13, 14). Specifically, betaine can be freely filtered in the kidney and reabsorbed into the circulation, so it is primarily excreted in sweat instead of urine (15, 16). Betaine accumulation depends on transporters, and it primarily distributes to the kidneys, liver, and brain (2). Although betaine is utilized in most tissues (such as the kidneys and brain) as an osmoprotectant, its primary role is to act as a methyl group donor in liver metabolism (17, 18).

### Betaine as an Osmoprotectant

In contrast to inorganic salts, osmoprotectants are highly soluble small organic compounds that accumulate in large amounts in cells without disrupting cell function; these compounds protect against osmotic stress (19). Hyperosmosis can cause water efflux and a concomitant reduction in cell volume; these effects are detrimental to cell survival (20). Thus, to balance hyperosmosis and protect cells from shrinkage and death, the accumulation of different types of osmoprotectants, such as betaine, sorbitol, and taurine, is essential (21–25). In contrast to other osmolytes and inorganic salts, such as urea and Na^+^, betaine reduces the ability of water molecules to solvate proteins, thus stabilizing the native protein structures (26). In addition, betaine can also increase the cytoplasmic volume and free water content of cells to prevent shrinkage in hyperosmotic conditions and to inhibit various hyperosmotic-induced apoptosis-related proteins (27, 28). Due to these advantages, additional betaine can be used to counter the pressure when tissues are hypertonic. For example, in the kidneys, hypertonicity increases the levels of the betaine-γ-aminobutyric acid (GABA) transport system (GAT4/BGT1) in the basolateral plasma membrane to obtain more betaine; however, under normal physiological conditions, BGT1 levels are low, and this transporter is present primarily in the cytoplasm in Madin–Darby canine kidney (MDCK) cells (21).

### Betaine as a Methyl Group Donor

Betaine is not only a metabolite of choline but also a methyl group donor that participates in methylation. Methylation, such as that of DNA and protein, is an essential biochemical process in animals. A previous study has shown that the availability of methyl group donors influences methylation levels (29). It has been acknowledged that betaine, methionine, and choline are the most important methyl group donors present in diets. Nevertheless, the major role of methionine is a substrate for protein synthesis, and choline contributes primarily to forming the cell membrane and neurotransmitters. The transmethylation reaction of betaine, which is part of a one-carbon metabolism *via* the methionine cycle, occurs principally in the mitochondria of liver and kidney cells. In this reaction, BHMT catalyzes the addition of a methyl group from betaine to homocysteine to form methionine, which is subsequently converted to dimethylglycine (DMG) (30). DMG has two available methyl groups and is possibly degraded to sarcosine and ultimately to glycine. Similarly, methionine synthase (MS), a vitamin B12-dependent enzyme, can also catalyze the formation of methionine from homocysteine with a donor methyl group from N5-methyltetrahydrofolate. These reactions are important in animals because they conserve methionine, detoxify homocysteine, which is a cause of cardiovascular disease (31), and produce S-adenosylmethionine (SAM) (32). SAM is generated from methionine *via* methionine adenosyltransferase (MAT), and SAM is a principal methylating agent. After demethylation, SAM is transformed into S-adenosylhomocysteine (SAH). The ratio of SAM:SAH affects various SAM-dependent methyltransferases, including protein-L-isoaspartate methyltransferase (PIMT), phosphatidylethanolamine methyltransferase (PEMT), protein arginine methyltransferase (PRMT), and isoprenylcysteine carboxyl methyltransferase (ICMT). These enzymes are associated with the protein repair progress, lipid metabolism, protein–protein interactions, and GTPase activity (33–38). One molecule of SAH is subsequently hydrolyzed by SAH hydrolase to form one homocysteine molecule and one adenosine molecule. Notably, this reaction is reversible, and the direction of the reaction depends on whether these products are removed. All of these reactions constitute the methionine cycle. Furthermore, with the help of cystathionine β-synthase, a vitamin B-6-dependent enzyme, homocysteine can be transformed into cystathionine *via* the transsulfuration pathway. In this pathway, homocysteine catabolism leads to an increase in the production of glutathione (GSH), taurine, and other metabolites (39–41). Dietary betaine supplementation has been demonstrated to have an impact on various sulfur amino acids (SAAs) (2). For example, such supplementation effectively increases the available methionine and SAM (42, 43). Therefore, betaine acts as a methyl donor and plays an influential role in SAA metabolism; the details of this metabolic pathway are shown in Figure 1B.

## Anti-Inflammatory Effects of Betaine on Diseases

Inflammation, an immune reaction, is an essential and primary process of host defense and wound healing. However, excessive or prolonged inflammation may become the pathogenesis of various diseases. Due to this, using natural compounds to treat diseases could be a good strategy by controlling the intensity of the inflammatory reaction. For instance, many studies show that GABA is anti-inflammatory (44). Consequently, using betaine in response to inflammation has sparked heated debate in recent years. Next, this review will discuss the primary mechanisms through which betaine exerts its anti-inflammatory effects on diseases.

### Betaine Ameliorates SAA Metabolism Against Oxidative Stress

Reactive oxygen species (ROS) are by-products of biological energy-generating reactions; in particular, they are produced in the mitochondria where oxidative metabolism primarily occurs. Under normal conditions, the body has two detoxification systems that can clear ROS and free radicals: antioxidant enzymes and antioxidant agents (45, 46). Catalase, superoxide dismutase (SOD), melatonin, and GSH are examples of these detoxification agents (47–49). However, excess ROS levels are a threat to cells because they alter the stability of nucleic acids, proteins, and the lipid membrane; furthermore, high ROS levels likely cause pathological processes, including inflammation (50).

Sulfur amino acids such as homocysteine, methionine, SAM, SAH, and cysteine are involved in various essential metabolic pathways, including GSH synthesis and protein synthesis, and transmethylation reactions. Although homocysteine contributes to GSH synthesis (51), various studies have demonstrated that hyperhomocysteinemia ultimately induces oxidative stress and apoptosis (52, 53). Betaine treatment can directly influence homocysteine concentrations *via* stimulating homocysteine to form methionine to regulate SAA concentrations. For example, ethanol-induced ROS and free radicals can suppress methionine synthase (MS) activity to inhibit remethylation and induce hyperhomocysteinemia (54). To compensate for this decrease in MS activity, betaine was used as an alternate methyl donor that improved BHMT activity to generate methionine and SAM and remove homocysteine in the livers of ethanol-fed Wistar rats (54, 55). However, it is worth noting that C57B6 mice showed a decrease or no change in BHMT expression, rather than a compensational increase (56). As betaine converts homocysteine to methionine, methionine concentrations are closely related with betaine. Methionine plays an important role in antioxidation. For example, methionine can reduce oxidative stress *via* chelation, and it can be used by hepatocytes for GSH synthesis (57, 58). In addition, this reaction is essential for generating SAM and removing homocysteine. Studies have demonstrated that SAM is a direct antioxidant in the body and that it can modulate GSH metabolism (59, 60). Moreover, based on the reversibility of the reaction that converts SAH to homocysteine and adenosine, homocysteine concentrations would further decrease (61). SAH is a powerful inhibitor of SAM-dependent methyltransferases; which methylate various compounds, such as nucleic acids and proteins (62). Kwon and colleagues found that betaine could significantly increase the SAM:SAH ratio and MAT activity (63). Kharbanda and colleagues found that betaine could prevent nitric oxide synthase 2(NOS2) expression; this process is initiated by inflammation, and the SAM:SAH ratio is increased to maintain NOS2 promoter methylation (64). In addition, homocysteine can also be converted to cysteine *via* the irreversible transsulfuration pathway, and cysteine then forms either taurine *via* cysteine dioxygenase (CDO) or GSH *via* γ-glutamylcysteine synthetase (65). Researchers found that betaine treatment inhibited CDO activity and decreased taurine levels, while increased the production of GSH to neutralize oxidative stress in AFLD and NAFLD mice (11, 63, 66).

Few studies indicated antioxidant enzymes, such as SOD 2 and glutathione S-transferases (GST), were changed after betaine treatment, but most results have shown no significant changes. Thus, more researches are needed in the future to confirm whether these antioxidant enzymes really participated in the process (55, 67, 68). Considering the above studies, the primary antioxidant mechanism of betaine may occur through ameliorating SAA metabolism. These changes after betaine treatment and the oxidation-related functions of primary SAAs are shown in Table 1.

**Table 1:** Changes in the oxidation-related functions of primary sulfur amino acids after betaine treatment.

Compound	Change	Functions	Reference
Methionine	Upregulated	GSH synthesis; reduces oxidative stress	(57, 58, 69)
S-adenosylmethionine	Upregulated	Increases cellular GSH content; antioxidant	(59, 60)
S-adenosylhomocysteine	Downregulated	Inhibits methyltransferases	(62)
		Induces oxidative stress	
Homocysteine	Downregulated	Induces oxidative stress; GSH synthesis	(51–53, 70)
Cysteine	Upregulated	GSH synthesis; reduces oxidative stress	(65)
GSH	Upregulated	Antioxidant	(71)

### Betaine Inhibits the NF-κB Signaling Pathway

The pathway of the transcription factor nuclear factor-κB (NF-κB) controls many genes involved in inflammation; these genes include the pro-inflammatory cytokines tumor necrosis factor-alpha (TNF-α), interleukin 1 beta (IL-1β) and interleukin 23 (IL-23). Therefore, it is not surprising that many inflammatory diseases involve chronically activation of NF-κB (72–74). Consequently, the NF-κB pathway has become an essential candidate for inflammation treatment. Researchers found that betaine can suppress NF-κB activity and various downstream genes (75–77). For example, in an early study of aged kidneys, betaine treatment suppressed NF-κB activity and the expression of a variety of related genes, including TNF-α, vascular cell adhesion molecule-1 (VCAM-1), intracellular cell adhesion molecule-1 (ICAM-1), inducible nitric oxide synthase (iNOS), and cyclooxygenase-2 (COX-2) (75). Notably, in this and another study about atherogenesis, the authors found that betaine inhibited NF-κB by suppressing two important activators, mitogen-activated protein kinases (MAPKs) and nuclear factor-inducing kinase/IκB kinase (NIK/IKK) (75, 76). NIK/IKK can relieve IκB inhibition and initiate the transcriptional activation of NF-κB (78). MAPKs consist of c-Jun NH2-terminal kinase (JNK), protein 38 (p38), and extracellular signal-regulated kinase (ERK1/2) and are involved in inflammation and the response to pro-inflammatory cytokine expression (79). Mechanistically, betaine exerts its effects by maintaining thiol levels, particularly GSH, to inhibit ROS production and NF-κB activity (80). Furthermore, betaine also inhibits some upstream signaling molecules that induce the activation of NF-κB. Classically, Toll-like receptors (TLRs) participate in an important upstream signaling event, which eventually culminates in activating NF-κB. In an *in vitro* study, betaine treatment prevented lipopolysaccharide (LPS, specific activator of TLR-4)-induced NF-κB activation in RAW 264.7 murine macrophage cells (81). Another study showed that betaine treatment improved hypothalamic neural injury *via* inhibiting the TLR-4/NF-κB signaling pathway to restore fructose-induced astrogliosis and inflammation. This study suggests that betaine can inhibit histone deacetylases 3 expression, which can activate NF-κB *via* binding to IκBα (82). Another study showed that betaine treatment can reduce the mRNA and protein expression levels of high-mobility group box 1, a positive regulator of TLR-4 activation to restrict inflammation (83). In addition, betaine can also reduce endogenous damage-associated molecular pattern (DAMP) generation to inhibit the NF-κB pathway. In conclusion, betaine has anti-inflammatory effects through its inhibition of NF-κB signaling pathway.

### Betaine Inhibits NLRP3 Inflammasome Activation

The leucine-rich family, pyrin-containing 3 (NLRP3) inflammasome is a large cytosolic protein complex that contains the nucleotide-binding domain, leucine-rich repeat-containing (NLR) family member NLRP3, the important adapter molecule ASC, and mature caspase-1. When TLRs recognize DAMPs or pathogen-associated molecular patterns, NF-κB can be activated to promotes mRNA expression of interleukin precursors, including pro-IL-18 and pro-IL-1β, as well as NLRP3 (84). The completely assembled NLRP3 inflammasome activates caspase-1 to mediate the production of mature IL-1β and IL-18, which are involved in initiating inflammation (85). It is important to ameliorate inflammatory reactions *via* inhibiting NLRP3 inflammasome activity.

Earlier studies have shown that betaine can directly increase heme oxygenase-1 expression levels in hepatocytes (86); this effect may suppress the NLRP3 inflammasome to protect against LPS-induced and d-galactosamine-induced inflammation in the liver (87, 88). Recent studies have demonstrated that betaine treatment can significantly inhibit NLRP3 inflammasome-related proteins, such as NLRP3 and mature caspase-1, and the levels of pro-inflammatory cytokines, including IL-1β, in a dose-independent manner in fructose-induced NAFLD models (82, 89, 90). The same phenomenon was found in betaine-treated db/db mice; this finding shows that the mechanism is associated with a forkhead box O1 (FOXO-1) inhibition of thioredoxin-interacting protein (TXNIP), which can promote the production of ROS to trigger NLRP3 inflammasome assembly (12). The FOXO family contains six members, including FOXO-1 and FOXO-6, that are found in mammals. The main role of FOXO factors is the regulation of cell growth, cell death, proliferation, differentiation, and oxidative stress response (91, 92). Activated FOXO-1 promotes TXNIP activity, which is the endogenous inhibitor of ROS-scavenging protein thioredoxin, resulting in producing more ROS (93). In addition, activated PKB/Akt can phosphorylate the active form of FOXO-1 to trigger its exit from the nucleus into the cytoplasm; this change makes FOXO-1 inactivation (94). In this study, betaine treatment increased the levels of PKB/Akt-mediated FOXO-1 phosphorylation. However, Kathirvel and colleagues noted that betaine did not directly activate PKB/Akt, and its mechanism may be the result of enhanced insulin receptor substrate 1 (IRS-1) phosphorylation (10). Thus, we suggest that betaine could enhance IRS-1 activity to activate PKB/Akt; then, the activated PKB/Akt would inhibit FOXO-1 activation, which restricts TXNIP to suppress NLRP3 inflammasome components to excise its anti-inflammation effects. Moreover, a study found that betaine mediated inhibition of NLRP3 inflammasome activation played a more important role than that of NF-κB in response to renal inflammation (90). Overall, the anti-inflammatory effects of betaine are closely associated with its inhibition of NLRP3 inflammasome activation.

### Betaine Regulates Energy Metabolism to Relieve Chronic Inflammation

Energy metabolism disorders can lead to various chronic diseases, including obesity and diabetes, which generally have a systemic low-grade inflammation (95). Thus, restoring normal metabolism is an essential step that contributes to mitigating inflammation. As various reports have reported, betaine has effects on both lipid and glucose metabolism (10, 96). Regarding lipid metabolism, excessive fat accumulation resulting from the imbalance of lipid transportation, synthesis, and oxidation is considered to be the culprit of many diseases. Many studies have demonstrated that various factors, such as high-fat diets, antibiotics exposure and ethanol consumption, could lead to such situations (11, 97).

Betaine treatment can restore the imbalance between synthesis and oxidation to help attenuate fat accumulation (97–99). Song and colleagues found that an increased hepatic AMP-activated protein kinase (AMPK) activity may be involved mechanistically (98). AMPK serves as both a principal cellular energy sensor and a vital metabolic homeostasis regulator; in fact, AMPK controls many genes, such as sterol regulatory element-binding protein-1c (SREBP-1c), acetyl CoA carboxylase (ACC), and fatty acid synthase (FAS). Activated AMPK can inhibit fatty acid synthesis and promote fatty acid oxidation *via* regulating the expression of these genes (100). Betaine can increase AMPK phosphorylation and then inhibit ACC activity as well as SREBP-1c and FAS expression (98). This result supports the finding of another study in which AMPK could directly phosphorylate SREBP-1c and SREBP-2 at Ser372 to inhibit their activities to reduce lipogenesis and lipid accumulation in diet-induced insulin-resistant mice (101). Furthermore, activated AMPK promotes glucose uptake *via* improving glucose transporter type 4 (GLUT-4) translocation; these findings suggest a beneficial effect on insulin resistance (102). Regarding the mechanism of AMPK activation, changing the AMP:ATP ratio in cells under normal conditions activates AMPK (103). However, hepatic AMPK activation can occur independently of the AMP:ATP ratio *via* adiponectin (104, 105). Interestingly, in another study from Song, betaine could restore abnormal adipokine levels in NAFLD, and it upregulated adiponectin and downregulated leptin and resistin in adipose cells to attenuate the dysregulated lipid metabolism. Similar effects of betaine are supported by another *in vitro* study in human adipocytes (106). These results imply that the upregulation of adiponectin may contribute to AMPK phosphorylation (107). In addition, because these adipokines play roles in inflammation, this normalizing process is anti-inflammatory (106). In addition to activating AMPK, betaine treatment could potentially influence other lipid metabolism-related factors. Earlier studies showed that betaine can reduce triglyceride accumulation in apolipoprotein B (apoB)-deficient mice *via* decreasing peroxisomal proliferator-activated receptor alpha (PPARα) methylation (108). In another study, betaine restricted PPARγ transcriptional activity *via* inhibiting FOXO-1 binding to the PPARγ promoter to reduce fat accumulation (109). In a recent study, not only PPARα but also hepatic liver X receptor α (LXRα) were upregulated when betaine restored fatty acid oxidation inhibition (89). Although the mechanism of how betaine activates LXRα remains unclear, it may be associated with a SAM-related enzyme PRMT-3, which can directly increase LXRα activity (38). In addition, in a study of cisplatin-induced nephrotoxicity, betaine inhibited lipid peroxidation *via* suppressing renal thiobarbituric acid-reactive substance activation, which is mostly initiated by oxidative stress (110). In addition to altering fat synthesis and oxidation, betaine treatment can ameliorate lipid transport. A study found that betaine maintains liver SAM:SAH ratios to enhance phosphatidylcholine synthesis and normalize very-low-density lipoprotein (VLDL) production *via* promoting PEMT activity (111), and another study found that betaine stimulates apoB gene expression to form more VLDL (112).

With respect to glucose metabolism, studies have demonstrated that insulin resistance is associated with inflammation (113, 114). Morgan and colleagues discovered that betaine supplementation could directly act upon the insulin pathway to improve NAFLD (10). A similar phenomenon has been found in another study of type 2 diabetes (12). In these studies, betaine significantly reduced ser473-phosphorylated PKB/Akt levels, but it increased IRS-1 phosphorylation and thr308-phosphorylated PKB/Akt levels. The PKB/Akt regulates systemic and cellular metabolism, mainly by mediating cell proliferation, differentiation, and survival, and it is required for insulin signaling (115, 116). Then, thr308-phosphorylated PKB/Akt could restrict FOXO-1 and glycogen synthase kinase-3α activities (115). The former can decrease the expression levels of phosphoenolpyruvate carboxy kinase to reduce hepatic gluconeogenesis, whereas the latter can increase glycogen synthesis (117, 118). To verify whether betaine can directly initiate PKB/Akt, the authors used a PI3K inhibitor, wortmannin, and found it hard to detect activated PKB/Akt; these results suggest that betaine may directly enhance IRS-1 phosphorylation rather than directly activate PKB/Akt (10). The mechanism of how betaine enhances IRS-1 phosphorylation to improve insulin resistance remains unclear. However, Iwasaki and colleagues recently reported that PRMT-1 can methylate heterogeneous nuclear ribonucleoprotein (hnRNPQ) and may be involved in insulin signaling (119–121). Mechanistically, PRMT-1 can catalyze the addition of a methyl group from SAM to hnRNPQ; this process results in internalization and lasting insulin receptor activation. Notably, SAM concentrations are associated with betaine. Therefore, these evidences speculated that betaine could improve the available SAM to generate more methylation hnRNPQ *via* PRMT-1 and thus PKB/Akt activation. Besides IRS-PKB/Akt signaling pathway, Chen and colleagues found that betaine treatment could reduce the protein levels of X-box-binding protein-1, an endoplasmic reticulum (ER) stress-related protein; this reduction is likely to enhance p38–MAPK and mammalian target of rapamycin activities and ultimately reduce hepatic gluconeogenesis and insulin resistance (89). Therefore, we conclude that betaine exerts its anti-inflammatory effects *via* restoring energy metabolism. These main metabolic pathways and key factors mediated by betaine treatment in chronic inflammation are shown in Table 2.

**Table 2:** Main metabolic pathways and genes/proteins influenced by betaine treatment in inflammation diseases.

Results	Main metabolic pathway	Gene/protein	Function of gene/protein	Reference
Lipid metabolism↑	AMPK pathway↑	ACC↑	Fatty acid synthesis	
		FAS↑	Fatty acid synthesis	(98, 100, 101)
		SREBP-1c↑	Fatty acid synthesis	

	Others	PPARα↑, PPARγ↑	Fatty acid oxidation	(108, 109)
			Fatty acid oxidation	
		LXRα↑	Fatty acid oxidation	(89)
		TBARS↓	Lipid peroxidation	(110)
		Apo B↑	Cholesterol transport	(111, 122)

Glucose metabolism↑	IRS-1/Akt pathway↑	IRS-1↑	Insulin sensitivity	(10, 12, 123, 124)
		FOXO-1↓	Gluconeogenesis	
		GSK3α↓	Inhibits glycogen synthesis	

	Others	XBP-1↓	Gluconeogenesis	(89, 125)
		GLUT-4↑	Glucose transport	(102)

*The upward arrows indicate promotion*.

*The downward arrows indicate inhibition*.

### Betaine Mitigates ER Stress and Apoptosis

Endoplasmic reticulum (ER) stress is caused by the abnormal assembly of proteins, as either misfolded or unfolded proteins, in the ER lumen (126). Various proteins, such as C/EBP homologous protein (CHOP) and glucose-regulated protein 78 (GRP78), are involved in ER stress, and both of these proteins are ER stress markers (127). Massive ER stress is undesirable and leads to cell apoptosis. Apoptosis is a type of cell death, and takes part in the pathogenesis of inflammatory diseases (128). Although apoptosis has extrinsic and intrinsic pathways, the final process is completed by caspase family proteins, especially caspase-3 (129).

As mentioned, betaine can directly influence the homocysteine pool, and it has been reported that hyperhomocysteine can induce misfolded proteins, ultimately leading to ER stress (70). According to the research by Cheng, betaine can stabilize homocysteine levels and inhibit GRP78 and CHOP levels as well as cell death (130). Likewise, in another study, betaine inhibited both GRP78 and CHOP and reduced JNK activation (107). Interestingly, the JNK pathway can directly phosphorylate multiple IRS-1 sites, including serine-307. These modifications prevent insulin-stimulating IRS-1 tyrosine phosphorylation, which leads to insulin resistance (10). In addition to ER stress, betaine also inhibits apoptosis. In a recent study of rheumatoid arthritis synovial fibroblasts, Gaur and colleagues found that transcription factor-3 (ATF-3), an apoptosis-related molecule, is downregulated by betaine (131). In addition, betaine can inhibit caspase family proteins. In an *in vitro* study, adding adenosine to hepatocytes increased hepatic SAH levels and caspase-3 activity, both of which would be inhibited by betaine treatment (132). The inhibition of caspase-3 by betaine is also found in cisplatin-induced nephrotoxicity (110). Furthermore, betaine significantly reduced caspase-8, caspase-9, and caspase-3/7 activity in human corneal epithelial cells and MDCK cells under hyperosmotic stress (28, 133). Thus, we would like to believe that the mitigation of ER stress and apoptosis by betaine is essential to its anti-inflammatory effects.

## Applications of Betaine in Human Diseases

Recently, the effects of natural and nontoxic substances on human diseases have attracted considerable attention. Researches have shown that betaine has beneficial effects in various human diseases, such as obesity, diabetes, cancer, and Alzheimer’s disease (134–141). Obesity results from excessive fat accumulation and has potentially negative effects on health. Obesity can lead to various secondary diseases, such as NAFLD. In animal studies, dietary betaine has been demonstrated to positively affect body fat (142, 143). However, few studies have focused on the effect of betaine on human obesity, and some of the results are contradictory. The current studies show that plasma betaine concentrations are inversely correlated with body fat percentages in adults; subjects with higher plasma betaine concentrations tended to have better fat profiles and distributions (134, 135). In a recent study, Gao and colleagues found that this positive correlation between plasma betaine concentrations and a better body composition existed in only males (144). Although this area deserves attention, there are few studies exploring the influence of betaine supplementation on obesity. In the studies of Schwab and Favero, betaine supplementation did not affect body composition (145, 146). However, in another study from Gao and colleagues, a large general population was analyzed, and a higher betaine intake was correlated with a better body composition (147). Similarly, other studies show that plasma betaine concentrations are inversely associated with human NAFLD, but the results of betaine supplementation are up for debate (148–152). Thus, in order to get the reliable result, more studies need to focus on this area from now on. In addition, many animal studies have shown that betaine is closely linked with diabetes (12, 153, 154). Diabetes leads to hyperglycemia due to impaired glucose metabolism (155). Different from its role in obesity, plasma betaine concentration is a poor predictor for diagnosing diabetes in humans (137, 156, 157). Nevertheless, plasma betaine concentration is likely linked with secondary diseases of diabetes, such as microangiopathy (158). Studies have shown that abnormal urinary betaine excretion is closely associated with diabetes (136–138), but its diagnostic value is lower than that of other substances, such as choline and DMG (159). Currently, only one study has investigated the effect of betaine supplementation on diabetes (160). Therefore, to identify whether betaine supplementation is effective, more systematic studies will be needed in the future. Various human studies have found that in addition to its association with metabolic diseases, betaine intake is associated with cancers, such as breast cancer, lung cancer, liver cancer, colorectal cancer, and nasopharyngeal carcinoma (139, 140, 161–163). In these studies, a higher betaine intake resulted in a lower risk of cancer. Furthermore, research has suggested that cancer incidence could be decreased by 11% by consuming choline plus betaine (100 mg/day) (164). However, in some studies, contradictory results have been found (165, 166). For example, Lee and colleagues found no association between colorectal cancer and betaine intake (165). So far, most of these have been case–control studies; to obtain reliable results, placebo-controlled intervention trials and prospective studies will be needed. Recently, a study has shown that betaine intervention could restore hyperhomocysteine, which is a hallmark of Alzheimer’s disease (141), and attenuate the inflammatory reaction in Alzheimer’s disease patients (167). This finding further extended the range of betaine applications in human diseases.

In summary, despite some contradictory results, we propose that betaine may have an application in treatment or ameliorating symptoms of various huaman inflammatory diseases because of betaine’s significant anti-inflammatory effects (168). Notably, human diseases are undoubtedly more complex than animal disease models; therefore, to take advantage of the beneficial effects of betaine, researchers should continue to explore its mechanism and effects in humans.

## Conclusion

**Figure 2:**
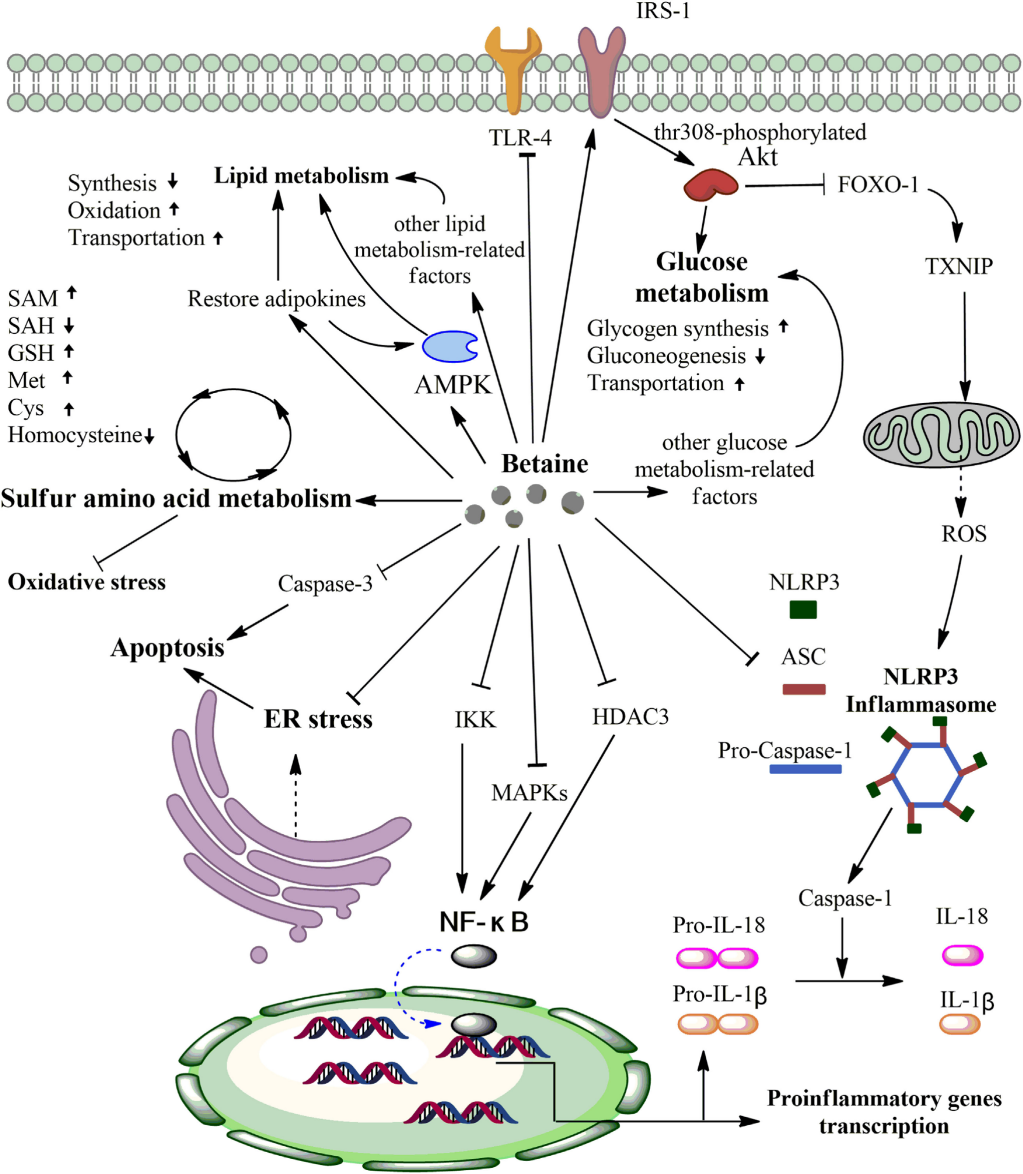
Primary anti-inflammatory mechanisms of betaine. First, betaine can alter various sulfur amino acid (SAA) concentrations *via* protecting SAA metabolism from oxidative stress. Second, betaine can inhibit IKK, MAPKs, HDAC3, and Toll-like receptor-4 (TLR-4) activities to downregulate the nuclear factor- κB (NF-κB) pathway and pro-inflammatory genes transcription. Third, betaine can reduce the expression levels of NLRP3 inflammation components (pro-caspase-1, ASC, and NLRP3) and inhibit the FOXO-1-induced NLRP3 inflammasome *via* enhancing the IRS/Akt pathway. Fourth, betaine significantly increases activated AMPK, restores adipokines that can activate AMPK, and activates other lipid metabolism-related factors to regulate lipid metabolism. Fifth, on the one hand, betaine increases phosphorylated IRS, which phosphorylates Akt at threonine 308, to improve glucose metabolism. On the other hand, betaine can influence other glucose metabolism-related factors to improve glucose metabolism. Sixth, betaine can inhibit caspase-3 to reduce apoptosis and repair endoplasmic reticulum (ER) stress. Akt, protein kinase B; AMPK, AMP-activated protein kinase; FOXO-1, forkhead box O1; TXNIP, thioredoxin-interacting protein; ROS, reactive oxygen species; IKK, nuclear factor-inducing kinase/IκB kinase; MAPKs, mitogen-activated protein kinases; HDAC3, histone deacetylases 3. SAM, S-adenosyl-L-methionine; SAH S-adenosyl-L-homocysteine; GSH, glutathione; Met, methionine; Cys, cysteine.

In conclusion, this review discusses the major physiological role of betaine as an osmoprotectant and a methyl group donor, as well as the anti-inflammatory effects of betaine in various diseases. These effects are primarily associated with protecting SAA metabolism from oxidative stress, inhibiting NF-κB and NLRP3 inflammasome activity, regulating energy metabolism, and mitigating ER stress and apoptosis (Figure 2). Although the data from animal experiments are compelling, the clinical situation appears to be much more complex than originally thought. For example, despite various animal studies reporting the effects of betaine supplementation and some mechanisms, human studies have shown contradictory results. Future studies should focus on both animal and clinical experiments to reduce errors from separate experiment types and to ensure the medicinal value of betaine. More importantly, it is worthwhile to further investigate betaine because its significant anti-inflammatory effects could be beneficial for treating inflammatory diseases.

## Author Contributions

YP, WR, and GZ designed the review article, and GZ wrote the review article. YP and WR revised the review article. CW, FH, NL, JD, GZ, and PL helped with designing figures and finding relevant literature. YP and WR approved the final manuscript.

## Conflict of Interest Statement

The authors declare that the research was conducted in the absence of any commercial or financial relationships that could be construed as potential conflicts of interest.
